# Management of skin necrosis and wound dehiscence following brown recluse spider bite in the course of breast reconstruction: A case report

**DOI:** 10.1016/j.jpra.2024.05.008

**Published:** 2024-05-31

**Authors:** Sten Kajitani, Kameron Reyes, Samiha Sajida, Mila Pastrak, Dr. Hisham Seify

**Affiliations:** a University College Cork, Ireland; b Orange Coast College, Costa Mesa, CA, USA; c Newport Plastics and Reconstructive Surgery, Newport Beach, CA, USA; d David Geffen School of Medicine, University of California, Los Angeles, CA, USA

**Keywords:** Breast reconstruction, Skin necrosis, Wound dehiscence, Brown recluse spider, Bite, Latissimus dorsi flap, Expander placement

## Abstract

**Background:**

Breast reconstruction following mastectomy is a critical component of breast cancer treatment, aimed at improving patient quality of life. However, the management is fraught with potential complications, including skin necrosis and wound dehiscence, which can significantly impact clinical outcomes.

**Case Presentation:**

We report a unique case of a patient, 5 years post-breast reconstruction following mastectomy and radiation therapy, who developed severe skin necrosis and wound dehiscence due to a brown recluse spider bite on the reconstructed breast. The complication necessitated the debridement of skin, removal of the implant, and further reconstruction with a latissimus flap.

**Discussion:**

The case underscores the unusual etiology of spider bite-induced necrosis in breast reconstruction and highlights the challenges and strategic considerations in managing such complications. Upon presentation, the patient's affected breast area showed signs of extensive necrosis and wound dehiscence, directly attributed to the cytotoxic effects of the brown recluse spider's venom. The venom's pathophysiology involves a complex cascade, leading to local and systemic effects. The local effects, marked by dermonecrosis, com- promised skin integrity in this instance. Systemic effects, not observed in this patient but potentially severe, can include hemolysis, coagulopathy, and acute renal failure, highlighting the seriousness of brown recluse spider bites.

**Conclusion:**

In conclusion, this case illustrates the complexities of managing breast reconstruction post-mastectomy complications, particularly those caused by external factors such as brown recluse spider bites. It highlights the need for meticulous attention to unusual etiologies of necrosis and dehiscence, demonstrating the importance of adaptable surgical strategies and a thorough understanding of venom pathophysiology in ensuring successful patient outcomes.

## Introduction

Breast reconstruction post-mastectomy is crucial for aesthetic restoration and quality of life in cancer survivors, yet it carries risks of complications like skin necrosis and wound dehiscence, which may require further surgery and modify the reconstruction approach. This report details a rare case where a patient, years after breast reconstruction and radiation therapy, suffered skin necrosis and wound dehiscence due to a brown recluse spider bite, highlighting a unique complication outside common postoperative issues.

Complications stemming from spider bites within the context of breast reconstruction are infrequently documented in medical literature.^1^, ^2^, ^3^ Spider bites are known for causing severe tissue necrosis mostly on the extremities and trunk.^4^ This unique case contributes to the understanding of external factors, like arachnid bites, as critical considerations in diagnosing skin necrosis and wound dehiscence in breast reconstruction.

## Case report

On March 08, 2017 the patient was diagnosed with right breast Ductal Carcinoma In Situ (DCIS) following mammography. Diagnostic confirmation was achieved through subsequent ultrasound and biopsy procedures within the same year.

The patient's reconstructive timeline commenced with bilateral mastectomy surgery and expander reconstruction on May 02, 2017, followed by radiation, completed Mid-January of 2018, and an implant placement in May 2018.

**Figure 1:**
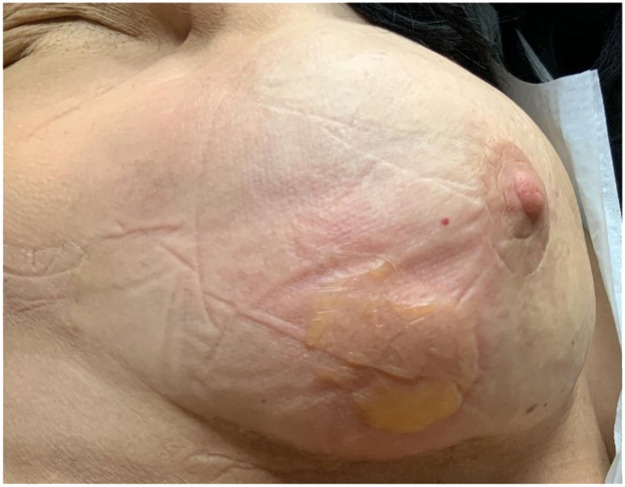
Initial bite presentation in November 2022 on day 0 after spider bite on right breast.

On November 28, 2022, the patient reported that she “felt something bite her while she was sleeping” associated with acute symptoms of irritation and blistering on the right breast area, with no evident signs of infection (Figure 1). Upon examination at the HOAG Emergency Room, laboratory tests and ultrasound results were negative for infection, effectively ruling out bacterial causes. The lesion, while initially uncharacterized, resembled a burn or insect bite in appearance, with no fluid accumulation detected in the surgical pocket nor indications of implant rupture on MRI. Conservative management commenced with local wound care and topical bacitracin; however, oral Trimethoprim/Sulfamethoxazole (TMP/SMX) was discontinued due to an adverse reaction from the patient.

**Figure 2:**
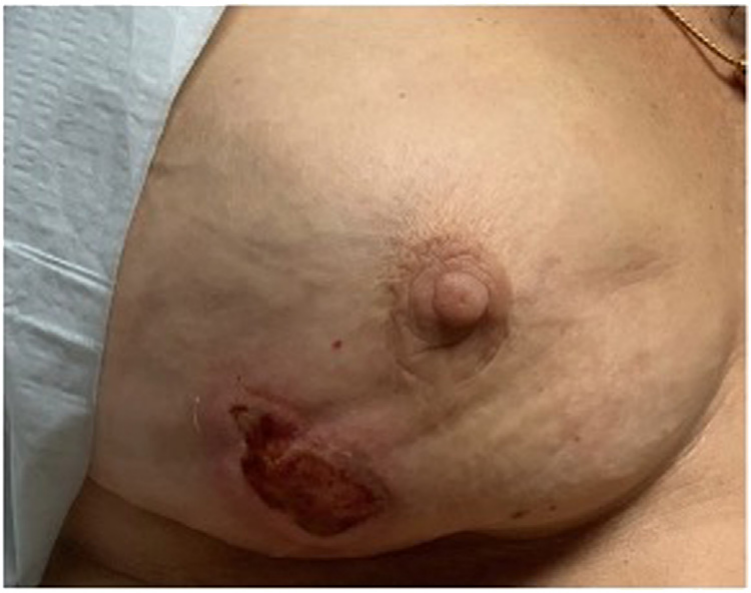
Development of a 5 cm (length) by 2.5 cm (width) necrotic lesion 1 month after initial presentation.

The diagnosis of a brown recluse spider bite was meticulously deduced based on the acute presentation of the lesion and the patient's account of feeling something bite her. The lesion's distinctive appearance, featuring a pale center surrounded by erythema, aligned with the hallmark signs of such envenomation.^1^^,^^4^^,^^5^ The lesion's progression to a 5 cm by 2.5 cm necrotic ulcer over the span of a month corroborated the expected timeline of necrosis development post-bite, typically manifesting no earlier than 7–14 days (Figure 2).^1^^,^^4^^,^^5^ The absence of significant systemic swelling or exudation, alongside these specific characteristics, reinforced the diagnosis of a brown recluse spider envenomation as the primary inciting factor.^1^^,^^4^^,^^5^

Crucially, the patient had previously undergone radiation therapy for breast cancer, which could be a differential for tissue damage in this patient. However, radiation damage typically presents as gradual thinning of tissues at the incision site, rather than acute adjacent tissue necrosis, as in this instance.^6^ Similarly, mechanical compression following radiation, another factor considered, tends to manifest gradually over time, and is more commonly associated with wound dehiscence along incision lines rather than adjacent tissue necrosis.^7^ However, radiation likely exacerbated the effects of the spider bite since it may have compromised the tissue's integrity, rendering it more susceptible to severe outcomes from the bite.^6^^,^^7^

**Figure 3:**
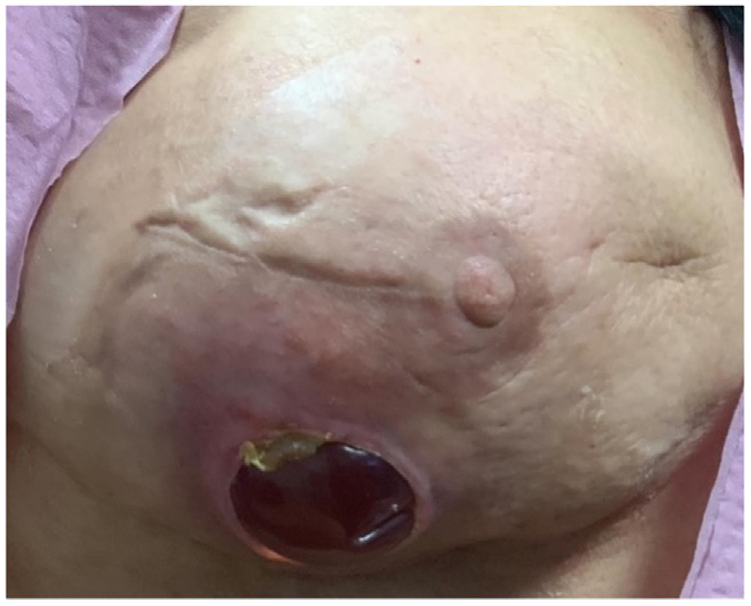
Perforation of the original breast implant through the dermis and epidermis secondary to necrosis from the brown recluse spider bite with evidence of surrounding tissue inflammation.

Hence, the acute nature of the bite, coupled with the patient's medical history of radiation therapy, provided a unique confluence of factors leading to the development of the necrotic lesion.^1^^,^^4^^,^^5^ On February 24, 2023, this culminated in a dehiscence wound that exposed the implant (Figure 3). This necessitated an immediate bilateral implant removal and capsulectomy alongside right breast reconstruction using a latissimus flap and expander.

**Figure 4:**
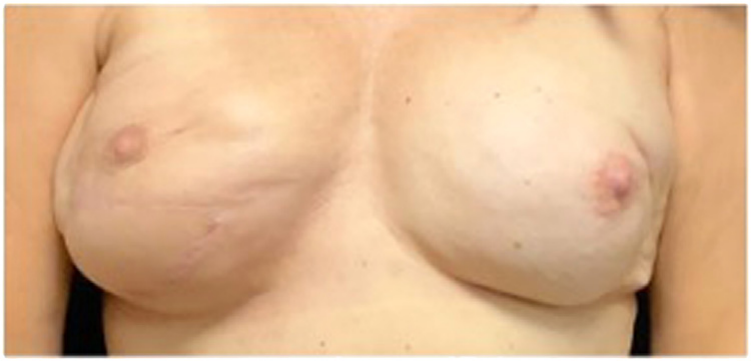
One-year postoperative photograph in February 2024 following breast reconstruction with placement of latissimus flap and expander (right breast).

Post-surgery, the patient's care was focused on wound management, monitoring for signs of infection or further necrosis, and ensuring the viability of the transposed flap. The expander allowed for gradual adjustment in breast volume, aligning with the patient's aesthetic goals and ensuring symmetry with the contralateral breast (Figure 4).^8^ Follow-up visits were scheduled to assess the healing process, expander adjustment, and the patient's satisfaction with the aesthetic outcome.

## Discussion

This case emphasizes the significance of addressing and managing intricate complications in breast reconstruction scenarios. Factors such as radiation, infection, and compromised blood supply impact the risk of necrosis and dehiscence, but a brown recluse bite adds a layer of complexity with its cytotoxic venom causing significant tissue damage.^1^^,^^4^^,^^5^

The management of such an unprecedented complication necessitates a nuanced understanding of both the pathophysiology of spider venom and the principles of breast reconstruction. The venom of the brown recluse spider contains a complex mixture of enzymes, including sphingomyelinase D, which leads to local and systemic effects.^5^ The local effects are characterized by dermonecrosis, which in this case, compromised the integrity of the skin.^1^^,^^4^^,^^5^ The systemic effects, although not prominently featured in this patient's course, can include hemolysis, coagulopathy, and acute renal failure, emphasizing the potential severity of brown recluse spider bites.^5^

The adoption of autologous tissue reconstruction alongside volume adjustment techniques via expanders for implants offers a comprehensive strategy for managing wound dehiscence and necrosis subsequent to envenomations.^8^ This methodology is consistent with established surgical practices aimed at mitigating tissue loss and enhancing the efficacy of reconstructive efforts.^1^^,^^4^^,^^5^^,^^8^ It further exemplifies a flexible approach capable of tackling diverse reconstructive challenges.

## Conclusion

In conclusion, this case report delineates a rare and complex complication of breast reconstruction post-mastectomy, emphasizing the unforeseen impact of a brown recluse spider bite leading to skin necrosis and wound dehiscence. Despite the rarity of such complications, the successful management of this case through the removal of implants, capsulectomy, and the innovative use of a latissimus flap with expander underscores the importance of flexibility in surgical planning and the capacity to address unique reconstructive challenges effectively.

## Compliance with ethical standards

Approval from ethics committee not required. All procedures were performed in compliance with relevant laws and institutional guidelines. The patient gives full informed consent to the publication of this case report.

## Funding

None.

## Declaration of competing interest

None.
